# Retroviral rebound syndrome after treatment discontinuation in a 15 year old girl with HIV attracted through mother-to-child transmission: case report

**DOI:** 10.1186/1742-6405-4-3

**Published:** 2007-02-23

**Authors:** Vanda Friman, Magnus Gisslén

**Affiliations:** 1 Department of Infectious Diseases, the Sahlgrenska Academy at Göteborg University, Sweden

## Abstract

A case of a 15 year old girl with retroviral rebound syndrome after discontinuation of highly active antiretroviral treatment (HAART) due to side effects is presented. The patient was transmitted with HIV at birth by her mother. She had recovered from severe AIDS after HAART was initiated five years earlier. This is the first case reported in the literature of retroviral rebound syndrome in a vertically transmitted HIV-infected patient.

## Introduction

Primary acute retroviral syndrome develop in more than 50% of patients infected with HIV [1,2] and more commonly when HIV is sexually transmitted than intravenously [3]. Acute retroviral syndrome has also been reported in children infected through breastfeeding [4], but there are no reports so far describing a similar seroconversion illness in children infected in utero or at birth.

In rare cases, symptoms similar to those of acute HIV infection can develop after antiretroviral treatment interruption [4-9]. The clinical presentations of this retroviral rebound syndrome, as well as of acute retroviral syndrome, often include fever, fatigue, pharyngitis, lymphadenopathy, rash and weight loss. Concomitant dramatic increases in plasma HIV RNA levels and decreases in CD4 cell counts are commonly observed. It has been unclear whether retroviral rebound syndrome could develop after treatment cessation also in patients infected with HIV since birth, given that acute retroviral syndrome is extremely rare, if at all exists, in vertically transmitted infants. We here report a case of retroviral rebound syndrome after cessation of highly active antiretroviral treatment (HAART) in a girl infected with HIV since birth via vertical transmission.

## Case report

A 15 year old girl with HIV since birth had to stop her antiretroviral treatment due to side effects. She started zidovudine monotherapy at the age of six, but continued to deteriorate clinically and immunologically during four years until she started HAART when it became available 1996. At the time she was hospitalized and severely ill with a *Mycobacterium avium intracellulare *sepsis. After treatment initiation with stavudine (30 mg QD), lamivudine (150 mg QD) and indinavir (600 mg TID) a remarkable recovery took place and her CD4-cell count increased from 10 to 410 in one year, and further to 920 × 10^6^/L during the following three years.

However, the TID dosage of indinavir was inconvenient and to render BID dosing possible, a ritonavir boosted regimen with indinavir (800 mg BID) and ritonavir (100 mg BID) was started about 1.5 months before treatment cessation. It was not known at that time (2001) that such high indinavir dosage very often resulted in nephrotoxic side effects, and the serum creatinine concentration increased from 59 to 132 μmol/L after the change. Consequently, her antiretroviral treatment was stopped and the creatinine concentration normalized again within two months.

**Figure 1:**
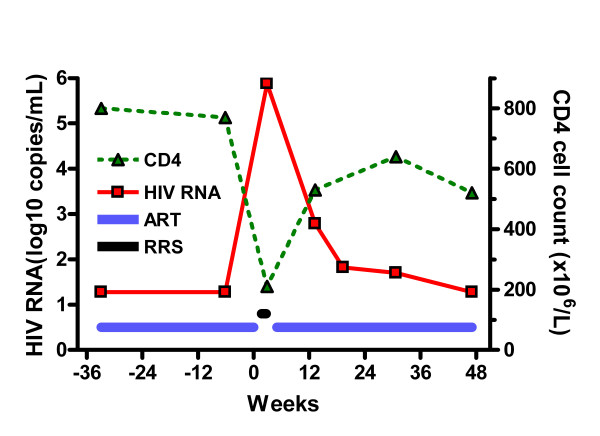
Changes in plasma HIV RNA and CD4 cell count before, during, and after antiretroviral cessation. Times on antiretroviral therapy (ART) and period of retroviral rebound syndrome (RRS) symptoms indicated.

Twelve days after the treatment discontinuation she presented with fever (39–39.5°C), lymphadenopathy, splenomegaly and abundant sweating during the nights. Her physical examination was normal and a chest radiography showed clear lung fields. Besides confirmed enlargement of the spleen, nothing abnormal was found with ultrasound or CT-scan of the abdomen. Blood cultures for bacteria, including mycobacteria, were negative. Serological testing for Epstein-Barr Virus (EBV), CMV and toxoplasmosis did not give any evidence of an ongoing infection. Routine laboratory showed discrete leucopenia and thrombocytopenia and slightly increased hepatic aminotransferase levels. Serum lactate and C-reactive protein were normal. Two weeks after treatment interruption the plasma HIV RNA level had increased from <50 copies/mL to >750000 copies/mL and the CD4 cell count decreased from 770 to 210 × 10^6^/L, figure 1.

Treatment with stavudine (30 mg BID), lamivudine (150 mg BID) and efavirenz (600 mg QD) was re-started just over one months after cessation, resulting in decreased HIV RNA and increased CD4 cell count again. The fever disappeared a few days before treatment was re-initiated.

## Discussion

Retroviral rebound syndrome has been described in several reports after discontinuation of successful antiretroviral treatment in patients with chronic HIV infection [5-9]. To our knowledge, this is the first description of this syndrome in a patient infected with HIV since birth. The clinical picture with mononucleosis-like symptoms, together with typical laboratory findings and profound rapid viral rebound reinforce the diagnosis. Retroviral rebound syndrome is a diagnosis of exclusion and syphilis, CMV and EBV-infections are important differential diagnosis. Serological analyses did, however, not give support to those infections in this case. Also other diseases with influenza-like symptoms may be considered as differential diagnoses to retroviral rebound syndrome, and although unlikely, another infection couldn't completely be excluded in our case.

The clinical presentation of retroviral rebound syndrome is mostly mild, although more severe manifestations have been described [7,9]. A dramatic rapid loss of peripheral CD4 cells was seen in our case and an increased risk for disease progression and serious illness have been shown in larger studies after structured treatment interruptions [10]. Thus, but also due to the risk of antiretroviral resistance development, structured treatment interruption strategies are not options to be used in the clinical setting. However, there are situations where cessation of treatment is inevitable, as for example by reason of severe side effects.

This case shows that retroviral rebound syndrome can develop after cessation of successful antiretroviral treatment also in patients infected with HIV since birth.

## Competing interests

The author(s) declare that they have no competing interests.

## Authors' contributions

VF and MG contributed to the patient care, data interpretation, and writing of the paper.
